# Fighting fire with fire: engineering a microbe into a therapeutic defense against drug-resistant biofilms

**DOI:** 10.1093/synbio/ysad008

**Published:** 2023-04-15

**Authors:** Charlotte Ayn Cialek

**Affiliations:** Inscripta Inc., Boulder, CO, USA

Bacteria use a bag of tricks to thwart attempted assault. One strategy involves forming a barrier called a biofilm that can both deflect antibiotics and block the human immune defense (1, 2). A bacterial colony shielded by a biofilm can persist and drastically increase the severity of an infection. Not all bacteria produce biofilms, but the ones that do cause >65% of human infections (1, 2).

*Pseudomonas aeruginosa* is a type of pathogenic bacteria prone to forming biofilms in human lungs and on synthetic surfaces, like ventilator breathing tubes. As such, it can cause recurring antibiotic-resistant pneumonia, leaving patients without good treatment options (3). Recently published in *Nature Biotechnology*, researchers in Dr. Maria Lluch-Senar’s lab at the Barcelona Institute of Science and Technology led the development of a new strategy to combat *P. aeruginosa*-based infections. They engineered a species of lung-dwelling bacteria able to dissolve the biofilm and then locally produce an antibiotic to kill the now-exposed pathogen (3). If further developed, this strategy could become the first living microbial cure for life-threatening *P. aeruginosa* respiratory infections. Using microbes as therapeutics is a strategy being explored and has shown promise for several other diseases (4, 5).

Conventional therapeutics involve producing, isolating and purifying a compound before administration to a patient. In contrast, therapeutic microbes bypass these steps since they produce and deliver the therapeutic molecules while living within the patient. From this principle, Mazzolini *et al.* generated a new type of therapeutic capable of fighting biofilm-based infections. Using genetic engineering, the scientists weaponized the lung-dwelling *Mycoplasma pneumoniae* strain CV2 against *P. aeruginosa.* To accomplish this, they added genes to CV2 to continuously produce and secrete massive quantities of glycoside hydrolases (PelAh54 and PslGh55) and an alginate lyase (A1-II′), which work together to deconstruct the polysaccharide and alginate structural components of biofilms. Additionally, they engineered CV2 to produce pyocin S5 or L1, which are *P. aeruginosa*–specific antibiotics (6, 7). This strategy enabled CV2 to both break down biofilms and poison *P. aeruginosa* with a one-two punch.

CV2’s parental strain, *M. pneumoniae*, is not completely harmless to humans since it can cause weak, non-biofilm lung infections. To attenuate CV2, the team removed pathogenic genes from *M. pneumoniae*, thus generating a safe strain incapable of causing infection or inflaming lung tissue in live mice. Further clinical testing will be needed to ensure the safety of this strain in humans.

After designing and building the CV2 strain, the authors tested its efficacy on multidrug-resistant *P. aeruginosa* biofilms that had naturally aggregated onto breathing tubes extracted from infected patients. Although the biofilms were resistant to antibiotics alone, they dissolved when cocultured with CV2. The exposed *P. aeruginosa* was then rapidly killed by the pyocin antibiotic expelled by CV2. The treatment worked, too, in live mice: disseminating aerosolized CV2 into the lungs of *P. aeruginosa*–infected mice resulted in animals with a better prognosis, reduced biofilms, less inflamed and damaged lung tissue, and reduced mortality. These promising results in mouse models could, in future studies, translate to human patients with biofilm-based respiratory infections.

Microbes have inherent capabilities that can be used or enhanced to fight diseases. As a microbe, CV2 could attack and outcompete the cleverest defenses of *P. aeruginosa*, a strategy of fighting fire with fire which is similar to natural microbial competition. Other microbial characteristics are also useful for therapeutic development, such as the natural tumor-targeting ability of *Escherichia coli* Nissle or its ability to produce and display a coating of active enzymes for enzyme replacement therapy. Although no microbial-based therapeutics have been approved, many are undergoing development, and some, including using microbes to fight colorectal cancer or treat enzyme deficiencies, have ongoing human clinical trials (4).

Undertaking novel microbial-based therapeutics like these brings a whole new series of inherent challenges, including dose control of the therapeutic compound, location-specific targeting and adaptation to a specific microenvironment of the human body. Since microbial-based therapeutics are alive, the engineered microbes will likely require biocontainment, such as a genetically encoded ‘kill-switch’, to remove them from the patient and prevent them from growing outside the patient or lab. Overcoming these challenges could give microbial-based therapeutics the potential to revolutionize some classes of therapeutics, such as the case of CV2, which could combat biofilm-based respiratory infections unlike any non-living therapeutic.
